# Comparative Efficacy and Safety of Chinese Herbal Injections Combined With Cyclophosphamide and 5-Fluorouracil Chemotherapies in Treatment of Breast Cancer: A Bayesian Network Meta-Analysis

**DOI:** 10.3389/fphar.2020.572396

**Published:** 2021-01-27

**Authors:** Shuyu Liu, Haojia Wang, Miaomiao Wang, Xiaohong Hu, Wenqian Yang, Ruiqi Jin, Yifei Geng, Mengwei Ni, Jiarui Wu, Xiaomeng Zhang

**Affiliations:** Department of Clinical Chinese Pharmacy, School of Chinese Materia Medica, Beijing University of Chinese Medicine, Beijing, China

**Keywords:** breast cancer, network meta-analysis, bayesian model, randomized controlled trials, Chinese herbal injections

## Abstract

**Background:** Given the limitations of chemotherapy for the treatment of breast cancer (BC) and the wide exploration of Chinese herbal injections (CHIs), this network meta-analysis (NMA) was conducted to analyze the comparative efficacy and safety of nine CHIs combined with CF (Cyclophosphamide and 5-Fluorouracil) chemotherapy regimens in the treatment of BC.

**Methods:** Several electronic databases were searched to identify randomized controlled trials (RCTs) from inception to January 6, 2020. RCTs were screened by pre-established eligibility criteria, and the quality of which was assessed using the Cochrane risk of bias tool. Outcomes such as the clinical effectiveness rate, performance status, peripheral hemogram, and detection of T-lymphocyte subsets were analyzed using the Winbugs 1.4.3 and Stata 13.0 software. Surface under the cumulative ranking curve (SUCRA) probability values were applied to rank the examined treatments. Cluster analysis was performed to compare the effect of CHIs between two or three different outcomes.

**Results:** A total of 84 RCTs involving 7855 patients and nine CHIs were included. The results showed that compared to CF chemotherapy regimens alone, the ones injected along with Aidi, Shenmai, Shenqi Fuzheng, Kangai, Kanglaite, or Shengmai combined with CF can improve the clinical effectiveness rate. Aidi, Shenmai, Shenqi Fuzheng, Compound Kushen, Kangai, and Kanglaite injection combined with CF can improve the performance status. Shenqi Fuzheng injection was considered as a favorable choice for relieving adverse reactions. According to the results of cluster analysis, Aidi injection and Compound Kushen injection plus CF were more favorable for the clinical effectiveness rate and performance status.

**Conclusion:** In conclusion, Shenqi Fuzheng, Compound Kushen, Aidi, and Kangai injection combined with CF chemotherapy regimen have more significant effects for patients with BC. However, more high-quality clinical RCTs, especialy which correctly use blinding and allocation concealment, are required to support the conclusions.

## Introduction

Breast cancer (BC) is one of the most common cancers and the main cause of cancer death in women around the world (Siegel et al., 2019). The high morbidity and high mortality of BC pose a huge potential threat to public health in China (Zheng et al., 2013; Fan et al., 2014; Chen et al., 2016). Although significant progress has been made in the fields of surgical treatment, endocrine therapy, chemotherapy, radiotherapy, and targeted therapy for BC in the past few decades, most BC patients still experience symptoms such as cancer metastasis, relapse, and adverse reactions (ADRs) (Wang and Li, 2018). In the chemotherapy of breast cancer, anthracyclines, such as doxorubicin (A), epirubicin (E) and pirarubicin (T), are the most widely used broad-spectrum anti-tumor drugs with a definite clinical effect. Therefore, this study selected CF chemotherapy regimens, especially those with anthracyclines as a combination medicine, as a control group for efficacy evaluation.

As an important part of complementary and alternative medicine, traditional Chinese medicine (TCM) has become one of the main methods of comprehensive anti-cancer treatment due to its advantages in treating complications and preventing drug resistance (Zhou et al., 2017). According to TCM theory, the basic pathogenesis of BC is meridian block, qi stagnation, blood stasis, etc. (Gao and Wang, 2013). Therefore, nourishing liver and kidney, enhancing the body's resistance and eliminating pathogens were deemed to be the principle of treating BC (Dong and Ren, 2018). Chinese herbal injections (CHIs) has the characteristics of obvious curative effect and high bioavailability (Lai et al., 2012). At present, CHIs combined with chemotherapy has been widely used in the treatment of malignant tumors, but there is still a lack of high-quality evidence-based medicine at home and abroad. The preliminary search found that many classic Meta-analysis have been published before to evaluate the safety and effectiveness of a single kind of CHI as an adjuvant treatment of breast cancer. However, they cannot horizontally compare and rank the curative effects of various CHIs. And some meta-analysis does not reach required information size to provide convincing results. Bayesian network meta-analysis (NMA) can have the advantages of combining direct and indirect evidence to compare multiple interventions. This method can increase the credibility of the evidence and select the optimal CHIs for the BC treatment. Therefore, this study used a NMA method to comprehensively evaluate the efficacy and safety of CHIs combined with CF (Cyclophosphamide and 5-Fluorouracil) chemotherapy regimen against BC.

## Methods

This study is reported in strict accordance with the standard format of the Preferred Reporting Items for Systematic Reviews and Meta-Analysis Specification: PRISMA Extension Statement specification (Hutton et al., 2015; Ge et al., 2017).

### Search Strategy

A computerized search of PubMed, the Cochrane library, embase, the China National Knowledge Infrastructure database (CNKI), Wanfang database, the China Science and Technology Journal database (VIP), and SinoMed databases for literatures on RCTs of CHIs in the treatment of BC was performed up to January 6, 2020. The searching strategy was developed with reference to the Cochrane Handbook for Systematic Reviewers (version 5.1.0). The search terms were divided into three parts: CHIs, BC, and RCTs. The varieties of CHIs searched included Shenqi Fuzheng injection (SQFZI), Compound Kushen injection (CKI), Shenmai injection (SMI), Kangai injection (KAI), Aidi injection (ADI), Kanglaite injection (KLTI), Huangqi injection (HQI), Huachansu injection (HCSI), and Shengmai injection (SI). The detailed search strategy taking PubMed as an example is described in Presentation File.

### Inclusion Criteria

#### Types of studies

Randomized controlled trials (RCTs) regarding CHIs combined with CF in the treatment of BC were eligible, which is referred to as “random”, with or without blinding.

#### Types of Participants

All patients were diagnosed with BC pathologically and histologically, no limitation on gender and nationality.

#### Types of Interventions

Patients in control group only received CF chemotherapy regimens, including CAF, CEF, CTF, CMF, FAC, FEC, etc., (C: cyclophosphamide; F: 5-fluorouracil; A: doxorubicin; T: pirarubicin; E: epirubicin; M: methotrexate). Patients in treatment group received CHIs with CF therapy.

#### Types of Outcomes

Primary outcomes include clinical effectiveness rate, performance status, T-lymphocyte subsets (including CD3 +, CD4 +, CD8 +, CD4 +/CD8 +), peripheral hemogram including white blood cells (WBC) and platelets (PLT), and ADRs. Secondary outcomes include tumor markers, the Karnofsky Performance Score (KPS), ECG changes, comparison of LVEF, liver and kidney function, and myocardial enzyme spectrum. According to the WHO Objective Response Criteria in Solid Tumors, The clinical effectiveness rate = [number of complete response (CR) patients + partial response (PR)]/total number of patients ×100%. In accordance with KPS functional status scoring criteria, there are three levels: improvement (KPS score increased by more than 10 points), stability (KPS score changed by less than 10 points) and decrease (KPS score decreased by more than 10 points). An increase of more than 10 points in KPS score is considered as a significant improvement in performance status. RCTs that have at least any one of the primary outcome indexes were included in this study.

### Exclusion Criteria

The exclusion criteria were as follows: 1) There are other TCM treatment methods except CHIs in the chemotherapy regimen; 2) For the repeatedly published articles, only remained the latest or more comprehensive ones; 3) Researches with incomplete data or obvious errors; 4) The article could not be obtained.

### Data Extraction and Quality Assessment

NoteExpress software was used to eliminate duplicate RCTs. Two researchers independently read the title and abstract of RCTs, and obviously irrelevant literatures were screened out. Then, the remaining articles were read in full according to the inclusion and exclusion criteria for rescreening. In case of any disagreement, it may be handed over to a third party for judgment. The following information were recorded from the included RCTs: the first author, publication year, patient characteristics (sample size, age, disease duration, TNM stage), specific chemotherapy regimen, usage and dosage of CHIs, course of treatment, outcomes, type of studies and the domains of risk of bias.

Two researchers evaluated the risk of bias of included RCTs using the Cochrane Handbook for Systematic Reviewers (version 5.1.0), RCT risk of bias assessment tool. Evaluation indicators include: 1) sequence generation (selection bias); 2) allocation concealment (selection bias); 3) blinding of patients and personnel (performance bias); 4) blinding of outcome assessors (detection bias); 5) incomplete outcome data (attrition bias); 6) elective reporting (reporting bias); 7) other bias. Each indicator contains three levels: low risk, unclear and high risk. Disagreement during the evaluation process may be referred to a third party for determination.

### Data Analysis

WinBUGS 1.4.3 software was used for statistical analysis of data. Binary outcomes were calculated as odds ratio (OR); continuous outcomes were calculated as weighted mean difference (MD), along with 95% confidence intervals (95% CIs) for effect sizes. OR excluding one or MD values excluding 0 were considered as statistically significant (Liu, 2012). Stata 13.0 software was applied to draw cumulative probability ranking plots, reticular relationship plots, and funnel plots for each intervention. The cumulative probability ranking plot can simultaneously obtain the surface under the cumulative ranking area curve (SUCRA). SUCRA with 100% indicated that the intervention was absolutely effective, and 0% indicated that the intervention was absolutely ineffective (Chen and Chen, 2013; Jia et al., 2015). Cluster analysis resulted in relatively better interventions in both cluster measures (Bian et al., 2011; Wu et al., 2017). In the network graph, the dot area represented the number of patients with relevant interventions, and the thickness of the line between each point represented the number of included studies (Leucht et al., 2013; Li et al., 2014). In the funnel plot, evenly distributed points indicated that the publication bias of the included RCTs was small (Gao et al., 2018). In addition, if there was a closed loop of intervention in the study, a consistency test was required. The results were expressed as *p* value, IF (inconsistency factor) and 95% CIs. If *p* value was greater than 0.05 and IF value was close to 0, the corresponding direct comparison evidence was consistent with indirect comparison evidence (Jinatongthai et al., 2017).

## Results

### Search Results

**Figure 1:**
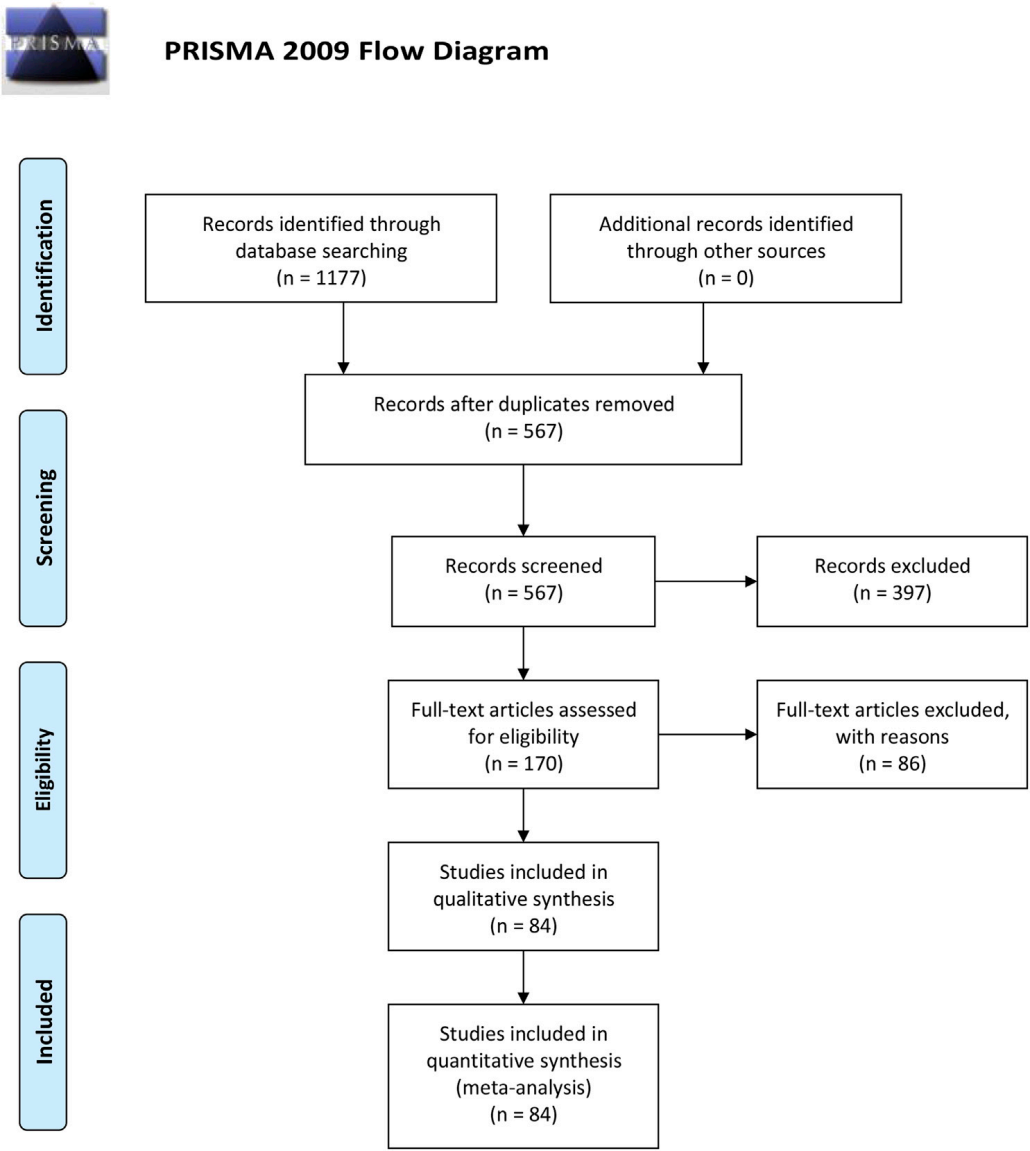
Prisma flow diagram.

As Figure 1 shown, a total of 1177 RCTs were retrieved according to the established searching strategies. After the exclusion of duplications and irrelevant studies, 170 RCTs were remained. After screening on full-text level, 86 RCTs were excluded for the following reasons: 1) not an RCT (*n* = 5); 2) Intervention was not met the inclusion criteria (*n* = 4); 3) disease was not BC (*n* = 11); 4) Outcomes were not in accordance with the inclusion criteria (*n* = 59); 5) duplicated data (*n* = 7). A total of 84 papers were eventually included, all of which were conducted in China from 1999 to 2019.

### Inclusion Studies and Characteristics

**Figure 2:**
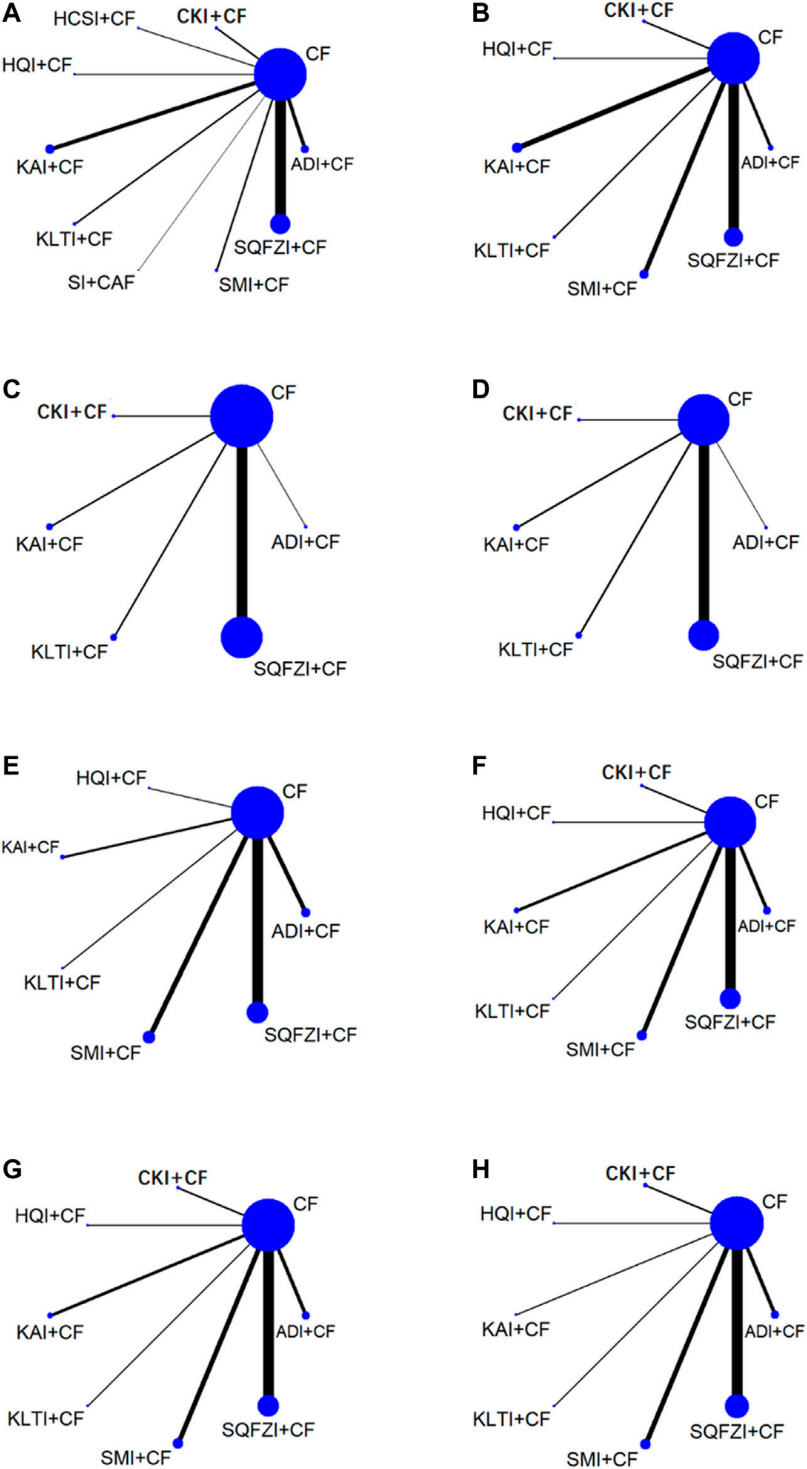
Network graph for different outcomes. **(A)** Clinical effectiveness rate; **(B)** Performance status; **(C)** Changes of peripheral blood leukocytes; **(D)** Peripheral blood platelet changes; **(E)** CD3^+^; **(F)** CD4^+^; **(G)** CD8^+^; **(H)** CD4+/CD8+. Note: SQFZI, Shenqi Fuzheng injection; CKI, Compound Kushen injection; SMI, Shenmai injection; KAI, Kangai injection; ADI, Aidi injection; KLTI, Kanglaite injection; HQI, Huangqi injection; HCSI, Huachansu injection; SI, Shengmai injection; CF, Cyclophosphamide and 5-Fluorouracil.

A total of 84 RCTs were finally included, including nine CHIs and 7855 female patients (4025 patients in the experimental group and 3830 patients in the control group), whose age ranged from 26 to 66 years old. The nine CHIs involved were ADI (9 RCTs), SMI (12 RCTs), SQFZI (33 RCTs), CKI (8 RCTs), HCSI (2 RCTs), HQI (2 RCTs), KAI (2 RCTs), KLTI (5 RCTs), and SI (1 RCTs). All of the nine CHIs were given via intravenous drip once a day, most of which were treated with a course of 21 days. Detailed information of included RCTs are shown in Table 1. The relationship of outcome indicators is shown in Figure 2.

**TABLE 1:** Characteristics of the studies included in this meta-analysis.

Study ID	Cases (E/C)	Average age	TNM clinical stage (E/C)				Treatment group intervention	Control group intervention	Course	Outcomes
			Ⅰ	Ⅱ	Ⅲ	Ⅳ				
Song et al. (2006)	48/48	E: 51 C: 50	NR				ADI 50 ml + CAF/TA	CAF/TA	21d × 2	①⑤
Yang (2005)	30/30	E: 48.4 C: 47.6	/	/	17/18	12/13	ADI 100 ml + CAF	CAF	21d × 2	①②③
Chen WM (2016)	39/40	E: 46.73 ± 14.29 C: 45.98 ± 15.78	/	/	29/28	10/12	ADI 100 ml + CEF	CEF	21d × 6	②③⑤⑥
Dang and Wang (2010)	28/20	E: 36.2 ± 3.6 C: 37.5 ± 4.2	5/4	19/14	4/2	/	ADI 100 ml + CTF	CTF	21d × 3	①④⑤⑦⑩
Liu et al. (2005)	50/50	45	Ⅱ-Ⅲ				ADI 100 ml + CEF	CEF	21d × 2	①③⑤⑥
Li and Gong (2006)	32/20	E: 46.2 ± 2.6 C: 44.5 ± 3.2	5/3	22/14	5/3	/	ADI 100 ml + CEF	CEF	21d × 3	①⑤⑦⑩
Ye et al. (2002)	18/17	E: 47.2 C: 47	NR				ADI 100 ml + CAF	CAF	15d	①②⑤
Ren and Zhang (2005)	60/40	39.5	Ⅱ-Ⅲ				ADI 80 ml + CMF	CMF	21d × 2	①⑤
Han et al. (2014)	64/64	46.7 ± 20.3	NR				ADI 100 ml + CEF	CEF	21d × 6	③⑤
Xiong et al. (2006)	40/43	E: 31–69 C: 33–71	NR				SMI 30 ml + CAF	CAF	10d	⑧
Wang et al. (2012)	31/30	E: 57.5 + 8.3 C: 60.3 + 6.9	NR				SMI 100 ml + CAF	CAF	21d	⑧
Yao et al. (2016)	49/49	30–65	NR				SMI 60 ml + CEF	CEF	21d × 6	⑨⑩
Chen et al. (2018)	45/45	E: 46.08 ± 4.90 C: 45.38 ± 4.97	12/10	21/23	12/12	/	SMI 50 ml + CTF	CTF	21d × 6	①⑩
Liu et al. (2014)	22/20	E: 50.18 ± 7.06 C: 48.62 ± 6.81	NR				SMI 100 ml + FAC	FAC	21d × 4	⑧⑨
Zhang (2013)	40/40	E: 26–62 C: 31–65	/	4/5	22/22	14/13	SMI 60 ml + CTF	CTF	21d × 2	①②③⑤⑦
Huang et al. (2009)	30/30	E: 41.2 C: 42.3	NR				SMI 60 ml + CTF	CTF	21d × 2	①②③⑤
Lao et al. (2011)	30/30	E: 52.4 C: 50.8	/	/	13/14	17/16	SMI 40 ml + CAF	CAF	14d × 2	②③⑤
Chen et al. (2010)	30/30	E: 46 C: 42	NR				SMI 100 ml + CAF	CAF	21d × 3	②③⑤
Liu et al. (2009)	30/30	E: 47 C: 46	/	/	11/13	19/17	SMI 60 ml + CAF	CAF	28d × 3	②③⑤
Bu and Zhang (2018)	42/41	48.3	NR				SMI 50 ml + CTF	CTF	21d × 6	⑨⑩
Yang (2008)	461/436	NR	NR				SMI 50 ml + CAF	CAF	21d × 6	⑤⑧⑩
Wang F (2015)	47/45	E: 42.85 ± 3.11 C: 41.96 ± 3.88	/	57	35	/	SQFZI 250 ml + CAF	CAF	21d × 2	①⑦
Liu (2017)	52/52	E: 41.58 ± 3.23 C: 40.41 ± 3.72	NR				SQFZI 250 ml + CAF	CAF	21d × 3	①⑤⑦
Qi et al. (2013)	26/20	52	NR				SQFZI 250 ml + CAF	CAF	21d × 2	①④
Zou et al. (2006)	32/32	52.5	NR				SQFZI + CTF	CTF	14d	③
Lu et al. (2010)	58/52	48.5	NR				SQFZI 250 ml + CAF	CAF	21d × 3	②④
Diao et al. (2018)	47/47	E: 51.68 ± 9.36 C: 52.94 ± 10.14	14/15	23/21	10/11	/	SQFZI 250 ml + FEC	FEC	21d × 6	①③
Song (2004)	21/25	E: 52 C: 58	/	18/22	3/3	/	SQFZI 250 ml + CMF	CMF	14d × 2	②⑤⑥
Chen and Lin (2007)	34/34	51	NR				SQFZI 250 ml + CEF	CEF	21d × 6	④⑤
Yang (2016)	40/40	E: 42.12 ± 1.33 C: 42.89 ± 1.21	16/16	16/15	8/9	/	SQFZI 250 ml + CEF	CEF	28d × 2	①③⑤
Liu and Song (2017)	80/80	E: 45.36 ± 3.37 C: 46.21 ± 9.83	NR				SQFZI 250 ml + CAF	CAF	21d	①⑦
Zhang et al. (2018)	64/64	E: 49.49 ± 18.21 C: 49.60 ± 18.36	/	22/27	27/14	15/13	SQFZI 250 ml + CAF	CAF	21d × 2	①②④
Guo and Hu (2018)	46/46	E: 33–66 C: 32–64	NR				SQFZI 250 ml + CAF	CAF	21d × 6	③④⑥
Li et al. (2015)	80/80	E: 48.53 ± 5.17 C: 48.42 ± 5.13	NR				SQFZI 250 ml + CAF	CAF	21d × 6	①⑤
Liang et al. (2014)	27/27	45.8 ± 2.3	Ⅲ-Ⅳ				SQFZI 250 ml + CTF	CTF	21d × 2	①②⑤
Jia et al. (2016)	50/50	E: 45.35 ± 10.02 C: 43.78 ± 9.18	NR				SQFZI 250 ml + CAF	CAF	21d × 2	②④
Xie (2014)	45/45	E: 54.4 ± 3.8 C: 52.8 ± 4.3	NR				SQFZI 250 ml + CAF	CAF	21d × 3	①⑤
Xiang (2019)	54/54	E: 45.26 ± 10.68 C: 45.32 ± 10.52	NR				SQFZI 250 ml + CAF	CAF	21d × 2	①④⑤
Huang et al. (2008)	30/30	E: 47 C: 46	NR				SQFZI 250 ml + CTF	CTF	21d × 2	①②③⑤
Li S (2018)	20/20	E: 41.2 ± 1.5 C: 41.5 ± 1.5	/	/	13/14	7/6	SQFZI 250 ml + CAF	CAF	21d × 3	①④⑦
Xiao (2005)	55/53	56.7	NR				SQFZI 250 ml + FEC	FEC	NR	②③④
Sun and Zheng (2005)	43/39	E: 58 C: 60	NR				SQFZI 250 ml + CTF	CTF	21d × 2	②④
Dai et al. (2007)	65/61	E: 45.5 ± 26.8 C: 46.1 ± 27.5	/	31/29	34/32	/	SQFZI 250 ml + CEF	CEF	28d × 2	①③⑤
Chen XC (2016)	42/42	E: 42.65 ± 8.27 C: 42.63 ± 8.24	NR				SQFZI 250 ml + CAF	CAF	21d × 2	①③⑤
He (2015)	38/38	E: 41.75 ± 2.77 C: 40.85 ± 5.12	NR				SQFZI 250 ml + CF	CF	21d	①
Mi (2011)	50/50	E: 42 C: 45	4/3	19/21	4/3	3/3	SQFZI 250 ml + AC/CAF/EC/CEF/TA/TE	AC/CAF/EC/CEF/TA/TE	21d × 4	②⑤
Ma et al. (2015)	36/36	NR	NR				SQFZI 250 ml + CAF	CAF	21d × 3	①②③⑤
Wang W (2015)	65/65	E: 46.3 ± 4.6 C: 47.4 ± 5.2	NR				SQFZI 250 ml + CAF	CAF	21d × 3	②④
Wang (2013)	38/38	E: 45.5 ± 9.8 C: 45.2 ± 9.8	/	25/26	12/13	/	SQFZI 250 ml + CAF	CAF	21d × 3	①⑤⑦
Fu (2014)	45/45	32–52	/	55	35	/	SQFZI 250 ml + FAC	FAC	21d × 3	⑤⑦
Yuan et al. (2008)	38/35	NR	Ⅱ-Ⅲ				SQFZI 250 ml + CAF	CAF	NR	③
Wang et al. (2006)	40/32	E: 45.2 ± 9.8 C: 46.7 ± 10.5	NR				SQFZI 250 ml + CEF	CEF	NR	②
Yang et al. (2007)	58/52	E: 48 C: 49	NR				SQFZI 250 ml + CAF	CAF	21d × 3	②④
Zhu et al. (2008)	32/24	E: 52.5 C: 51	11/9	11/16	5/4	/	SQFZI 251 ml + CEF	CEF	21d × 4	③④
Wang and Liu (2007)	30/30	42.5	NR				CKI 20 ml + CTF	CTF	21d × 3	②⑤
Zhang and Zhang (2019)	45/45	E: 41.21 ± 1.02 C: 41.25 ± 1.45	NR				CKI 20 ml + CAF	CAF	21d × 6	①
Zhai (2014)	61/62	E: 42.7 ± 10.5 C: 43.5 ± 11.2	3/1	48/53	10/8	/	CKI 20 ml + CAF	CAF	21d × 6	③④⑤⑦
Sun (2009)	29/30	49.6	NR				CKI 30 ml + CAF	CAF	21d × 6	③
Ren et al. (2010)	62/60	39–65	NR				CKI 30 ml + CAF	CAF	21d × 6	②⑤⑥
Li (2012)	30/30	49.3 ± 0.9	/	8	31	21	CKI 15 ml + CTF	CTF	21d × 2	①⑤⑦
Wei (2010)	12/12	52	NR				CKI 20 ml + CTF	CTF	21d × 2	③⑦⑧
Gu et al. (2015)	45/44	E: 47.2 ± 2.4 C: 47.8 ± 3.1	12/13	19/18	14/13	/	CKI 15 ml + FEC	FEC	21d	①
Pan (2011)	80/80	56	Ⅲ-Ⅳ				HCSI 20 ml + CAF	CAF	14d × 2	①⑤
Song (2002)	26/21	50	/	12/16	9/8	1/1	HCSI 20 ml + CAF	CAF	28d	①⑤
Lu (2010)	30/30	E: 46.2 ± 9.3 C: 44.31 ± 1.1	NR				HQI 40 ml + CAF	CAF	14d × 2	①⑤
Huang et al. (2013)	30/30	E: 47.8 ± 3.2 C: 51.2 ± 3.5	NR				HQI 40 ml + CEF	CEF	21d × 2	①②③⑤
Shi et al. (2017)	30/30	E: 41.83 ± 4.31 C: 42.04 ± 4.25	1/2	23/21	6/7	/	KAI 30 ml + CAF	CAF	21d × 3	③④⑤
Su et al. (2016)	53/53	35–70	NR				KAI 60 ml + CTF	CTF	28d × 3	①⑤
Tan et al. (2018)	30/30	E: 54.1 ± 6.2 C: 51.2 ± 4.6	/	19/19	11/11	/	KAI 60 ml + CTF	CTF	21d × 3	①②⑤
Chen FW (2016)	25/25	E: 47.52 ± 10.31 C: 48.06 ± 10.28	NR				KAI 60 ml + TXT+CEF Sequential	TXT+CEF Sequential	21d	①②⑤
Li et al. (2006)	42/40	E: 58 C: 60	NR				KAI 40 ml + CTF	CTF	21d × 2	①②
Wu and Wu (2011)	30/30	E: 51.5 C: 50.5	NR				KAI 40 ml + CTF	CTF	21d × 2	①②⑤
Qiu (2016)	60/60	E: 45.8 ± 10.3 C: 46.7 ± 10.8	/	48/46	12/14	/	KAI 40 ml + CEF	CEF	21d × 4	①③⑤
Pan et al. (2008)	15/15	E: 56.62 C: 59.74	NR				KAI 60 ml + CEF	CEF	21d	②③⑤
Wu (2010)	49/47	E: 52.2 ± 11.2 C: 50.7 ± 10.5	/	37/37	12/10	/	KAI 40 ml + CAF	CAF	21d × 4	②④
Cao et al., 2009	156/80	45	Ⅱ-Ⅲ				KAI 60 ml + CEF	CEF	21d × 3	①⑤
Wang and Wang (2011)	20/20	46	NR				KAI 40 ml + CTF	CTF	21d × 2	②⑤
Zhu and Sun (2012)	30/30	E: 52 ± 5 C: 54 ± 3	/	17/19	11/13	/	KAI 40 ml + CAF	CAF	21d × 4	①
Pan et al. (2016)	45/45	E: 45.66 ± 7.92 C: 46.83 ± 7.85	/	/	25/26	20/19	KLTI 200 ml + CAF	CAF	21d × 6	③④⑥
Fu et al. (2015)	60/60	E: 46.7 ± 7.8 C: 47.5 ± 7.1	NR				KLTI 200 ml + CAF	CAF	21d × 6	①④⑥
Yin et al. (1999)	15/17	52	3	13	16	/	KLTI 200 ml + FACT	FACT	21d × 2	①②⑤
Gan et al. (2009)	37/40	E: 45 C: 46	16/18	21/22	/	/	KLTI 100 ml + CEF	CEF	21d × 2	①②⑤
Jiang et al. (2017)	55/55	E: 33–66 C: 35–66	/	/	30/31	25/24	KLTI 200 ml + CAF	CAF	21d × 6	⑥
Chen (2005)	45/43	E: 35–61 C: 35–59	NR				SI 40 ml + CAF	CAF	21d	①

Note: E, Treatment group; C, Control group; NR, Not reported; SQFZI, Shenqi Fuzheng injection; CKI, Compound Kushen injection; SMI, Shenmai injection; KAI, Kangai injection; ADI, Aidi injection; KLTI, Kanglaite injection; HQI, Huangqi injection; HCSI, Huachansu injection; SI, Shengmai injection; C, cyclophosphamide; F, 5-fluorouracil; A, doxorubicin; T, pirarubicin; E, epirubicin; M, methotrexate; ①: Clinical effectiveness rate; ②: Performance status; ③: T-lymphocyte subsets; ④: Peripheral hemogram; ⑤: ADRs; ⑥: Tumor markers; ⑦: KPS; ⑧: ECG changes; ⑨: Comparison of left ventricular ejection fraction; ⑩: Liver and kidney function and myocardial enzyme spectrum.

## Methodological Quality

**Figure 3:**
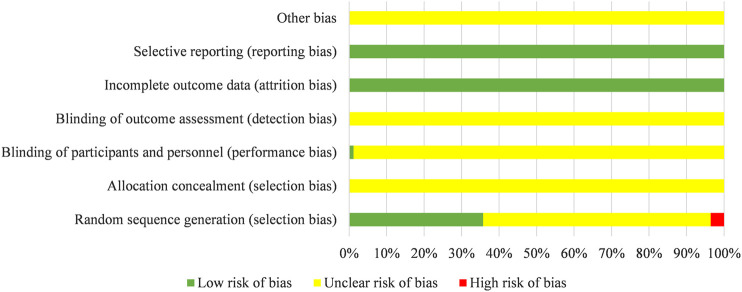
Assessment of risk of bias.

There were 22 studies of 84 included studies were grouped according to the method of random number tables, five studies described a method of randomization including draw lots or the envelope, two studies used a method of randomization including coin toss, and one study was divided into groups by stratified random method. The above 30 studies were rated as “low risk” regarding sequence generation. Three studies were evaluated as “high risk”, because they grouped according to the time of admission. The risk of remaining studies was deemed “unclear”, because they just mentioned “randomization”. One study adopted double-blind randomized clinical trial and evaluated for “low risk” regarding performance bias. The attrition bias of all studies were evaluated as “low risk” due to the lack of incomplete data. The risk of bias entries for the rest studies were rated as “unclear” due to insufficient information. Results are shown in Figure 3.

### Network Meta-Analysis

#### Clinical Effectiveness Rate

As the main outcome index, the clinical effectiveness rate directly reflected the curative effect of patients. A total of 47 studies referred to the clinical effectiveness rate, involving nine CHIs and 10 interventions. There were seven studies on ADI combined with CF, three studies on SMI combined with CF, 18 studies on SQFZI combined with CF, three studies on CKI combined with CF, two studies on HCSI combined with CF, two studies on HQI combined with CF, eight studies on AKI combined with CF, three studies on KLTI combined with CF, and one study on SI combined with CF. ORs showed that comparing to using CF alone, combined with ADI (OR = 0.43, 95%CIs: 0.30–0.75), SMI (OR = 0.47, 95%CIs: 0.27–0.93), SQFZI (OR = 0.45, 95%CIs: 0.33–0.55), KAI (OR = 0.43, 95%CIs: 0.31–0.65), KLTI (OR = 0.27, 95%CIs: 0.15–0.57), SI (OR = 0.18, 95%CIs: 0.03–0.48) on the basis of chemotherapy can improve the clinical effectiveness rate and make the difference between groups statistically significant. Specific values are shown in Table 2.

**TABLE 2:** Statistical results of network meta-analysis for the main outcomes (OR/MD value, 95% CI).

	Clinical effectiveness rate^a^	Performance status^a^	WBC	PLT	CD3+	CD4+	CD8+	CD4+/CD8+
**ADI + CF vs**								
SMI + CF	0.83 (0.45, 1.99)	0.61 (0.29, 1.78)	—	—	**−10.77 (−20.72, −0.18)**	−3.62 (−13.24, 7.48)	−3.62 (−13.24, 7.48)	0.18 (−0.96, 1.35)
SQFZI + CF	0.98 (0.62, 1.88)	0.72 (0.30, 1.79)	−0.77 (−5.09, 3.38)	−39.97 (−92.09, 2.38)	**−15.78 (−25.26, −6.78)**	−8.45 (−16.90, 0.77)	−8.44 (−16.90, 0.77)	−0.14 (−1.09, 0.95)
CKI + CF	0.79 (0.34, 2.02)	0.42 (0.18, 1.58)	−1.32 (−7.28, 5.69)	**−70.1 (−130.90, −0.81)**	—	**−11.7 (−21.27, −1.38)**	**−11.7 (−21.27, −1.38)**	0.21 (−0.91, 1.24)
HCSI + CF	0.84 (0.41, 1.81)	—	—	—	—	—	—	—
HQI + CF	0.66 (0.31, 1.77)	0.59 (0.17, 2.96)	—	—	−5.38 (−24.93, 13.34)	−1.29 (−18.56, 15.91)	−1.29 (−18.56, 15.91)	0.18 (−1.66, 1.94)
KAI + CF	0.95 (0.58, 1.91)	0.68 (0.24, 1.99)	−0.36 (−7.97, 6.19)	**−52.04 (−88.72, −17.52)**	**−25.71 (−39.01, −14.05)**	−8.95 (−19.29, 2.64)	−8.95 (−19.29, 2.64)	0.03 (−1.23, 1.11)
KLTI + CF	1.53 (0.67, 3.61)	0.77 (0.17, 3.04)	−0.62 (−4.63, 3.82)	−9.90 (−62.70, 29.80)	−13.97 (−50.11, 15.71)	−9.18 (−38.32, 21.07)	−9.18 (−38.32, 21.07)	0.07 (−2.47, 2.52)
SI + CF	2.45 (0.80, 14.95)	—	—	—	—	—	—	—
CF	**0.43 (0.30, 0.75)**	**0.21 (0.09, 0.47)**	−0.61 (−4.28, 3.57)	**−36.03 (−71.43, −1.37)**	−5.52 (−11.63, 1.74)	−1.43 (−8.54, 6.36)	−1.43 (−8.54, 6.36)	0.42 (−0.52, 1.35)
**SMI + CF vs**								
SQFZI + CF	1.06 (0.62, 2.28)	1.15 (0.63, 2.07)	—	—	−4.72 (−15.36, 5.33)	−4.85 (−13.72, 3.95)	−4.85 (−13.72, 3.95)	−0.31 (−0.98, 0.45)
CKI + CF	0.9 (0.39, 2.38)	0.71 (0.31, 1.97)	—	—	—	−8.30 (−18.26, 2.07)	−8.30 (−18.26, 2.07)	0.02 (−0.81, 0.77)
HCSI + CF	1.02 (0.37, 2.21)	—	—	—	—	—	—	—
HQI + CF	0.78 (0.29, 1.98)	1.00 (0.29, 4.29)	—	—	4.91 (−12.64, 24.80)	2.16 (−14.55, 18.14)	2.16 (−14.55, 18.14)	0.01 (−1.67, 1.46)
KAI + CF	1.12 (0.56, 2.36)	1.04 (0.52, 2.56)	—	—	**−14.43 (−29.33, −1.55)**	−5.31 (−16.26, 5.06)	−5.31 (−16.26, 5.06)	−0.16 (−1.10, 0.71)
KLTI + CF	1.85 (0.66, 4.32)	1.17 (0.39, 4.28)	—	—	−2.65 (−38.50, 26.13)	−5.50 (−34.17, 23.61)	−5.50 (−34.17, 23.61)	−0.20 (−2.24, 2.20)
SI + CF	2.9 (0.84, 16.52)	—	—	—	—	—	—	—
CF	**0.47 (0.27, 0.93)**	**0.32 (0.20, 0.55)**	—	—	5.53 (−2.58, 13.63)	—	2.14 (−5.45, 9.31)	0.24 (−0.30, 0.79)
**SQFZI + CF vs**								
CKI + CF	0.86 (0.40, 1.60)	0.61 (0.28, 1.49)	−0.64 (−4.66, 4.92)	−31.32 (−81.55, 44.85)	—	−3.39 (−11.96, 5.47)	−3.39 (−11.96, 5.47)	0.32 (−0.49, 0.97)
HCSI + CF	0.91 (0.38, 1.53)	—	—	—	—	—	—	—
HQI + CF	0.75 (0.30, 1.46)	0.88 (0.25, 3.39)	—	—	−12.51 (−40.08, 11.11)	6.87 (−9.52, 23.22)	6.87 (−9.52, 23.22)	0.34 (−1.30, 1.64)
KAI + CF	1.03 (0.62, 1.58)	0.91 (0.50, 1.97)	0.55 (−4.24, 4.82)	−12.51 (−40.08, 11.11)	−9.54 (−24.02, 1.68)	−0.43 (−9.92, 8.63)	−0.44 (−9.92, 8.63)	0.15 (−0.77, 0.95)
KLTI + CF	1.61 (0.72, 2.98)	1.06 (0.31, 3.06)	0.14 (−1.24, 1.64)	28.07 (−25.15, 86.27)	1.41 (−32.78, 31.08)	−0.60 (−30.01, 28.50)	−0.60 (−30.01, 28.50)	0.12 (−1.93, 2.65)
SI + CF	2.45 (0.88, 14.72)	—	—	—	—	—	—	—
CF	**0.45 (0.33, 0.55)**	**0.29 (0.20, 0.39)**	0.1 (−0.72, 1.20)	2.79 (−21.01, 24.86)	**10.41 (4.02, 17.07)**	**6.91 (2.03, 11.94)**	**6.91 (2.03, 11.94)**	**0.54 (0.06, 0.97)**
**CKI + CF vs**								
HCSI + CF	1.09 (0.40, 2.55)	—	—	—	—	—	—	—
HQI + CF	0.86 (0.30, 2.40)	1.42 (0.34, 5.75)	—	—	—	10.32 (−7.29, 27.01)	10.32 (−7.29, 27.01)	−0.01 (−1.56, 1.32)
KAI + CF	1.25 (0.56, 2.57)	1.51 (0.58, 3.80)	1.01 (−5.63, 6.79)	14.43 (−68.54, 71.25)	—	2.92 (−7.64, 13.19)	2.92 (−7.64, 13.19)	−0.18 (−1.08, 0.79)
KLTI + CF	1.99 (0.72, 4.83)	1.7 (0.51, 5.49)	0.76 (−5.04, 4.90)	58.15 (−1.22, 121.40)	—	2.58 (−27.23, 32.86)	2.58 (−27.23, 32.86)	−0.17 (−2.22, 2.17)
SI + CF	2.81 (0.95, 24.22)	—	—	—	—	—	—	—
CF	0.52 (0.27, 1.04)	**0.45 (0.20, 0.95)**	0.73 (−4.90, 4.77)	31.67 (−51.02, 86.36)	—	**10.26 (3.18, 17.41)**	**10.26 (3.18, 17.41)**	0.21 (−0.28, 0.81)
**HCSI + CF vs**								
HQI + CF	0.77 (0.32, 2.35)	—	—	—	—	—	—	—
KAI + CF	1.08 (0.60, 2.63)	1.11 (0.23, 4.27)	—	—	—	—	—	—
KLTI + CF	1.79 (0.76, 4.86)	1.09 (0.29, 5.64)	—	—	—	—	—	—
SI + CF	3.01 (0.90, 18.46)	—	—	—	—	—	—	—
CF	0.49 (0.29, 1.05)	0.33 (0.09, 1.06)	—	—	—	—	—	—
**HQI + CF vs**								
KAI + CF	1.42 (0.63, 3.33)	—	—	—	**−20.35 (−41.72, −0.31)**	−7.41 (−24.69, 9.68)	−7.41 (−24.69, 9.68)	−0.16 (−1.60, 1.50)
KLTI + CF	2.29 (0.83, 6.05)	—	—	—	−7.96 (−48.46, 27.45)	−8.12 (−40.43, 25.51)	−8.12 (−40.43, 25.51)	−0.14 (−2.45, 2.31)
SI + CF	**3.65 (1.02, 23.06)**	—	—	—	—	—	—	—
CF	0.61 (0.30, 1.37)	—	—	—	0.41 (−17.65, 18.33)	−0.07 (−15.61, 15.98)	−0.07 (−15.61, 15.98)	0.25 (−1.05, 1.74)
**KAI + CF vs**								
KLTI + CF	1.61 (0.60, 3.33)	1.14 (0.38, 3.85)	−0.43 (−4.61, 4.59)	40.8 (−5.46, 85.73)	10.96 (−25.85, 43.02)	−0.16 (−30.01, 30.23)	−0.16 (−30.01, 30.23)	0.02 (−2.14, 2.41)
SI + CF	2.41 (0.88, 15.02)	—	—	—	—	—	—	—
CF	**0.43 (0.31, 0.65)**	**0.31 (0.16, 0.52)**	−0.38 (−4.55, 4.40)	**16.28 (9.17, 24.62)**	**20.34 (10.66, 32.26)**	7.39 (−0.31, 15.29)	7.39 (−0.31, 15.29)	0.39 (−0.30.1.21)
**KLTI + CF vs**								
SI + CF	1.63 (0.46, 12.60)	—	—	—	—	—	—	—
CF	**0.27 (0.15, 0.57)**	**0.27 (0.10, 0.69)**	−0.03 (−1.02, 1.06)	−24.81 (−68.40, 22.39)	8.24 (−19.14, 43.95)	7.54 (−21.51, 36.57)	7.54 (−21.51, 36.57)	0.38 (−1.84, 2.41)
**SI + CF vs.**								
CF	**0.18 (0.03, 0.48)**	—	—	—	—	—	—	—

^a^ Note: indicates that the result is OR; Bold results indicate statistically significant differences between groups; SQFZI, Shenqi Fuzheng injection; CKI, Compound Kushen injection; SMI, Shenmai injection; KAI, Kangai injection; ADI, Aidi injection; KLTI, Kanglaite injection; HQI, Huangqi injection; HCSI, Huachansu injection; SI, Shengmai injection; CF, Cyclophosphamide and 5-Fluorouracil.

**Figure 4:**
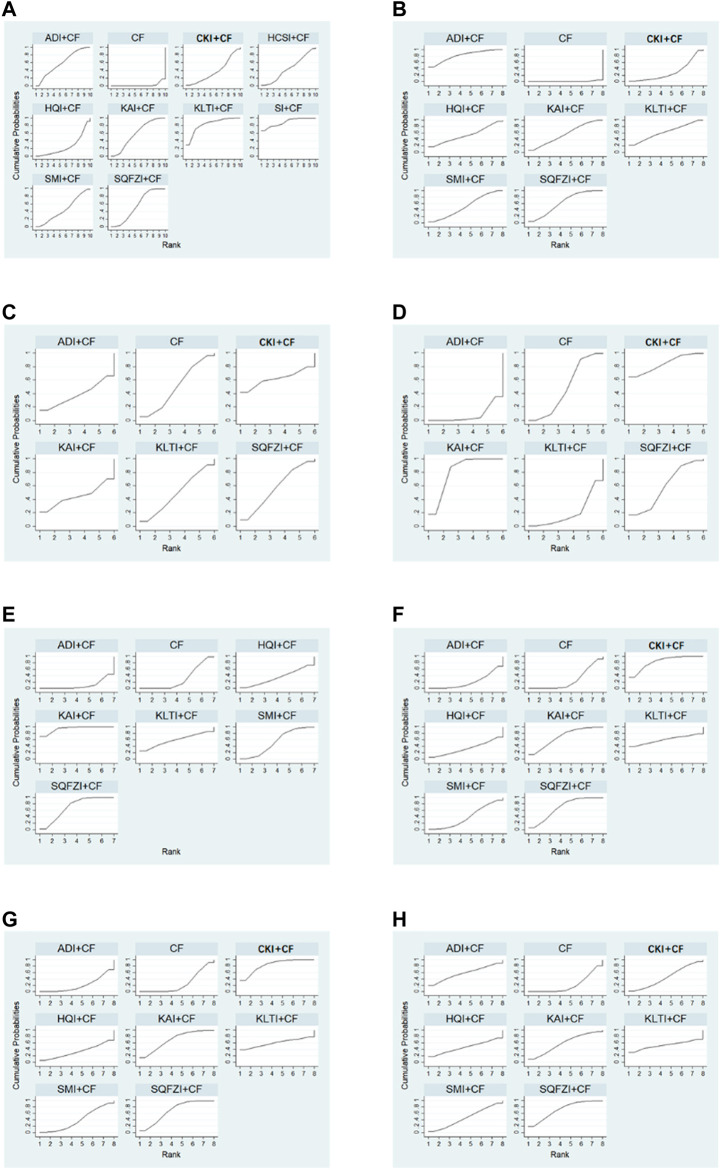
Plot of the surface under the cumulative ranking curves for all treatments. **(A)** Clinical effectiveness rate; **(B)** Performance status; **(C)** Changes of peripheral blood leukocytes; **(D)** Peripheral blood platelet changes; **(E)** CD3^+^; **(F)** CD4^+^; **(G)** CD8^+^; **(H)** CD4+/CD8+. Note: SQFZI, Shenqi Fuzheng injection; CKI, Compound Kushen injection; SMI, Shenmai injection; KAI, Kangai injection; ADI, Aidi injection; KLTI, Kanglaite injection; HQI, Huangqi injection; HCSI, Huachansu injection; SI, Shengmai injection; CF, Cyclophosphamide and 5-Fluorouracil.

After the ranking of each intervention efficacy, the combination of CF and SI (88.9%) had the highest probability of being the best treatment for BC in terms of improving the clinical effectiveness rate, followed by the combination of CF and KLTI (83.6%) and the combination of CF and ADI (59.2%). The ranking results of interventions are shown in Figure 4, and the SUCRA values are shown in Table 3.

**TABLE 3:** Surface under the cumulative ranking probabilities (SUCRA) results of main outcomes.

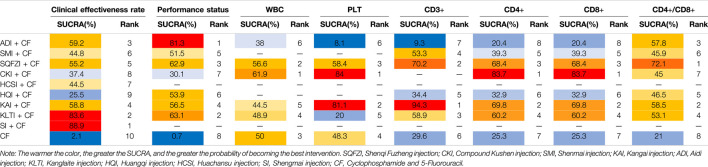

#### Performance Status

A total of 30 studies reported performance status, involving seven CHIs and eight intervenions. Three of the studies regarding ADI combined with CF, five regarding SMI combined with CF, 11 regarding CKI combined with CF, two regarding CKI combined with CF, one regarding HQI combined with CF, six regarding KAI combined with CF, and two of the studies regarding KLTI combined with CF. ORs showed that comparing to using CF alone, combined with ADI (OR = 0.21, 95%CIs: 0.09–0.47), SMI (OR = 0.32, 95%CIs: 0.20–0.55), SQFZI (OR = 0.29, 95%CIs: 0.20–0.39), CKI (OR = 0.45, 95%CIs: 0.20–0.95), KAI (OR = 0.31, 95%CIs: 0.16–0.52) as well as KLTI (OR = 0.27, 95%CIs: 0.10–0.69) on the basis of chemotherapy can improve the performance status and make the difference between groups statistically significant. Specific values are shown in Table 2.

After the ranking of each intervention efficacy, the combination of CF and ADI (81.3%) had the highest probability of being the best treatment for BC in terms of improving the performance status, followed by the combination of CF and KLTI (63.1%) and the combination of CF and SQFZI (62.9%). The ranking results of interventions are shown in Figure 4, and the SUCRA values are shown in Table 3..

#### Peripheral Hemogram

The report of peripheral hemogram included in this study mainly detected the changes of WBC and PLT. A total of 18 studies mentioned the changes of WBC, involving five CHIs and six interventions (ADI + CF, one RCT; SQFZI + CF, 12 RCTs; CKI + CF, one RCT; KAI + CF, two RCTs; KLTI + CF, two RCTs). MDs showed that in improving WBC decline in BC patients, compared with CF alone, combining with ADI, SQFZI, CKI, KAI or KLTI on the basis of chemotherapy made no difference between groups. A total of 15 studies mentioned PLT, involving five CHIs and six interventions. Comparing to using CF alone, combined with ADI (MD = −36.03, 95%CIs: -71.43∼-1.37), KAI (MD = 16.8, 95%CIs: 9.17–24.62) on the basis of chemotherapy can improve PLT decline in patients and make the difference between groups statistically significant. Specific values are shown in Table 2. Meanwhile, the combination of ADI and CF compared with the combination of CKI and CF (MD = −70.1, 95%CIs: −130.90∼−0.81), with the combination of KAI and CF (MD = −52.04, 95%CIs: −88.72∼−17.52), the latter two were more effective in improving PLT decline in BC patients and the difference between groups were statistically significant.

After the ranking of each intervention efficacy, the combination of CF and CKI (61.9%) had the highest probability of being the best treatment for BC in terms of improving WBC decline, followed by the combination of CF and SQFZI (56.6%). The combination of CF and CKI (84%) had the highest probability of being the best treatment for BC in terms of improving PLT decline, followed by the combination of CF and KAI (81.1%) and the combination of CF and SQFZI (58.4%). The ranking results of intervention measures are shown in Figure 4, and the SUCRA values are shown in Table 3.

#### T-Lymphocyte Subsets

The report of T-lymphocyte subsets in this study mainly involved four aspects: CD3+, CD4+, CD8+ and CD4+/CD8+. A total of 22 studies referred to CD3+, invoving six CHIs and seven interventions (ADI + CF, four RCTs; SMI + CF, five RCTs; SQFZI + CF, nine RCTs, HQI + CF, one RCT; KAI + CF, two RCTs; KLTI + CF, one RCT). A total of 22 studies mentioned CD4+ and CD8+, invoving seven CHIs and eight interventions (ADI + CF, four RCTs; SMI + CF, five RCTs; SQFZI + CF, 11 RCTs; CKI + CF, two RCTs; HQI + CF, one RCT; KAI + CF, three RCTs; KLTI + CF, one RCT). A total of 22 studies mentioned CD4+/CD8+, invoving seven CHIs and eight interventions (ADI + CF, four RCTs; SMI + CF, five RCTs; SQFZI + CF, 11 RCTs; CKI + CF, two RCTs; HQI + CF, one RCT; KAI + CF, one RCT; KLTI + CF, one RCT). MD values showed that, comparing to using CF alone, combined with SQFZI (MD = 10.41, 95%CIs: 4.02–17.07) and KAI (MD = 20.34, 95%CIs: 10.66–32.26) on the basis of chemotherapy can improve the decline of immune function of CD3+ in BC patients and make the difference between groups statistically significant. Combined with SQFZI (MD = 6.91, 95%CIs: 2.03–11.94) and CKI (MD = 10.26, 95%CIs: 3.18–17.41) on the basis of chemotherapy can improve the decline of CD4+ and CD8+ in BC patients and make the difference between groups statistically significant. Combined with SQFZI (MD = 0.54, 95%CIs: 0.06–0.97) on the basis of chemotherapy can improve the decline of CD4+/CD8+ in BC patients and make the difference between groups statistically significant. Specific values are shown in Table 2. Above outcomes showed that CF alone can inhibit the body’s immune function, while the combination of CF and CHIs can reduce the inhibition of immune function caused by CF.

After the ranking of each intervention efficacy, the combination of CF and KAI (94.3%) had the highest probability of being the best treatment for BC in the respect of reducing the decline of CD3+, followed by the combination of CF and SQFZI (70.2%) and the combination of CF and KLTI (58.9%). The combination of CF and CKI (83.7%) had the highest probability of being the best treatment for BC in the respect of reducing the decline of CD4+ and CD8+, followed by the combination of CF and KAI (69.8%) and the combination of CF and SQFZI (68.4%). The combination of CF and SQFZI (72.1%) had the highest probability of being the best treatment for BC in the respect of reducing the decline of CD4+/CD8+, followed by the combination of CF and KAI (58.5%) and the combination of CF and ADI (57.8%). The ranking results of interventions are shown in Figure 4, and the SUCRA values are shown in Table 3.

#### Adverse Reactions

Only one study clearly reported that no ADRs occurred during the study, and 53 studies had ADRs during the study. The ADRs mainly included five aspects: gastrointestinal reaction, abnormal renal and liver function, hair loss, nausea and vomiting, and decreased WBC.

A total of 12 studies reported gastrointestinal reactions, involving four kinds of CHIs. The result of NMA showed that CF combined with ADI (OR = 2.17, 95%CIs: 1.03–4.79), SQFZI (OR = 5.32, 95%CIs: 2.50–9.50), and KAI (OR = 2.34, 95%CIs: 1.24–4.79) can make statistically significant difference compared with using CF alone. SQFZI combined with CF was better than HCSI combined with CF (OR = 7.43, 95%CIs: 1.19–34.61) in the respect of relieving gastrointestinal reactions (Table 4).

**TABLE 4:** Statistical results of network meta-analysis for ADR outcomes (OR value, 95% CI).

	Gastrointestinal reactions	Abnormal renal and liver function	Hair loss	Decreased WBC	Nausea and vomiting
**ADI + CF vs**					
SMI + CF	—	—	0.40 (0.02, 6.29)	—	1.74 (0.39, 12.74)
SQFZI + CF	0.42 (0.16, 1.26)	0.36 (0.15, 1.07)	0.54 (0.02, 8.44)	1.63 (0.67, 3.57)	2.19 (0.41, 15.25)
CKI + CF	—	0.37 (0.10, 1.50)	—	1.50 (0.43, 6.13)	2.51 (0.48, 19.13)
HCSI + CF	2.99 (0.47, 16.06)	0.82 (0.05, 7.03)	—	—	
HQI + CF	—	—	0.60 (0.01, 24.53)	1.22 (0.29, 3.53)	1.95 (0.33, 15.80)
KAI + CF	0.92 (0.33, 2.66)	0.45 (0.14, 1.63)	0.35 (0.02, 6.00)	1.48 (0.67, 3.35)	2.87 (0.46, 24.95)
KLTI + CF					3.62 (0.43, 34.55)
CF	**2.17 (1.03, 4.79)**	1.40 (0.71, 3.46)	1.43 (0.11, 17.83)	**4.15 (2.28, 7.32)**	**6.84 (1.73, 51.08)**
**SMI + CF vs**					
SQFZI + CF	—	—	1.38 (0.13, 9.24)	—	1.14 (0.48, 2.89)
CKI + CF	—	—	—	—	1.35 (0.51, 3.74)
HCSI + CF	—	—	—	—	—
HQI + CF	—	—	1.55 (0.08, 27.36)	—	0.98 (0.31, 3.10)
KAI + CF	—	—	0.91 (0.12, 5.39)	—	1.47 (0.51, 4.34)
KLTI + CF	—	—	—	—	1.89 (0.41, 8.92)
CF	—	—	3.62 (0.99, 14.46)	—	**3.74 (2.18, 7.77)**
**SQFZI + CF vs**					
CKI + CF	—	1.15 (0.21, 3.76)	—	0.94 (0.26, 3.72)	1.17 (0.45, 3.73)
HCSI + CF	**7.43 (1.19, 34.61)**	2.11 (0.14, 15.74)	—	—	—
HQI + CF	—	—	1.15 (0.06, 29.40)	0.71 (0.20, 2.35)	0.86 (0.26, 2.83)
KAI + CF	2.20 (0.76, 5.19)	1.21 (0.37, 3.24)	0.67 (0.09, 6.15)	0.90 (0.42, 1.95)	1.31 (0.44, 3.48)
KLTI + CF	—	—	—	—	1.65 (0.37, 7.72)
CF	**5.32 (2.50, 9.50)**	**3.89 (1.94, 7.90)**	2.66 (0.61, 17.08)	**2.49 (1.57, 4.41)**	**3.31 (1.87, 6.03)**
**CKI + CF vs**					
HCSI + CF	—	2.07 (0.12, 18.66)	—	—	—
HQI + CF	—	—	—	0.77 (0.12, 3.83)	0.74 (0.20, 2.51)
KAI + CF	—	1.18 (0.24, 5.36)	—	0.99 (0.22, 3.55)	1.10 (0.33, 3.61)
KLTI + CF	—	—	—	—	1.37 (0.29, 6.84)
CF	—	**3.52 (1.27, 12.66)**	—	2.74 (0.71, 9.13)	**2.86 (1.27, 6.11)**
**HCSI + CF vs**					
HQI + CF	—	—	—	—	—
KAI + CF	0.30 (0.06, 1.88)	0.59 (0.07, 7.61)	—	—	—
CF	0.69 (0.17, 3.76)	1.73 (0.28, 25.26)	—	—	—
**HQI + CF vs**					
KAI + CF	—	—	0.60 (0.03, 10.65)	1.26 (0.34, 4.10)	1.53 (0.38, 6.12)
KLTI + CF	—	—	—	—	1.91 (0.34, 11.86)
CF	—	—	2.36 (0.19, 34.91)	**3.49 (1.01, 11.48)**	**3.84 (1.45, 10.60)**
**KAI + CF vs**					
KLTI + CF	—	—	—	—	1.28 (0.24, 6.16)
CF	**2.34 (1.24, 4.79)**	**3.16 (1.38, 9.00)**	**3.97 (1.19, 15.89)**	**2.80 (1.67, 5.19)**	**2.57 (1.10, 6.10)**
**KLTI + CF vs**					
CF	—	—	—	—	2.02 (0.48, 8.44)

Note: Bold results indicate statistically significant differences between groups; SQFZI, Shenqi Fuzheng injection; CKI, Compound Kushen injection; SMI, Shenmai injection; KAI, Kangai injection; ADI, Aidi injection; KLTI, Kanglaite injection; HQI, Huangqi injection; HCSI, Huachansu injection; SI, Shengmai injection; CF, Cyclophosphamide and 5-Fluorouracil.

A total of 19 studies reported abnormal renal and liver function, involving five kinds of CHIs. NMA showed that CF combined with SQFZI (OR = 3.89, 95%CIs: 1.94–7.90), CKI (OR = 3.52, 95%CIs: 1.27–12.66), and KAI (OR = 3.16, 95%CIs: 1.38–9.00) can make statistically significant difference compared with using CF alone (Table 4).

A total of 13 studies reported hair loss, involving five kinds of CHIs. NMA showed that CF combined with KAI (OR = 3.97, 95%CIs: 1.19–15.89) can make statistically significant difference compared with using CF alone (Table 4).

A total of 21 studies reported decreased WBC, involving five kinds of CHIs. NMA showed that CF combined with SMI (OR = 4.15, 95%CIs: 2.28–7.32), SQFZI (OR = 2.49, 95%CIs: 1.57–4.41), HQI (OR = 3.49, 95%CIs: 1.01–11.48), and KAI (OR = 2.80, 95%CIs: 1.67–5.19) can make statistically significant difference compared with using CF alone (Table 4).

A total of 19 studies reported nausea and vomiting, involving seven kinds of CHIs. NMA showed that, CF combined with ADI (OR = 6.84, 95%CIs: 1.73–51.08), SMI (OR = 3.74, 95%CIs: 2.18–7.77), SQFZI (OR = 3.31, 95%CIs: 1.87–6.03), CKI (OR = 2.86, 95%CIs: 1.27–6.11), HQI (OR = 3.84, 95%CIs: 1.45–10.60), and KAI (OR = 2.57, 95%CIs: 1.10–6.10) can make statistically significant difference compared with using CF alone (Table 4).

The results of area under the adverse reaction curve showed that, SQFZI combined with CF had the highest likelihood of being the best treatment for improving the gastrointestinal reaction (96.7%) and abnormal renal and liver function (77.8%) in BC patients, KAI combined with CF had a better effect on the symptoms of hair loss (73.5%), SMI had more advantages in reducing decreased WBC (80.9%), and ADI combined with CF was more effective in reducing nausea and vomiting (82.8%). The details are shown in Table 5.

**TABLE 5:** Surface under the cumulative ranking probabilities (SUCRA) results of ADR outcomes.

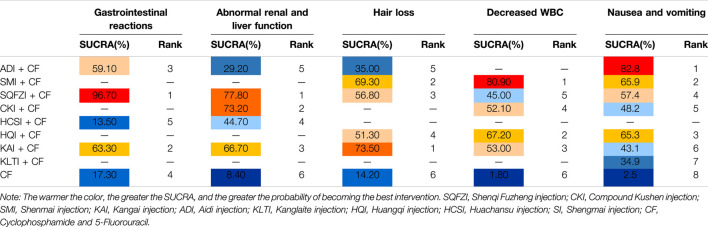

In 53 studies that reported ADRs, interventions of the control group were all CF, and other ADRs involved were reported as follows. ADI combined with CF was used as a treatment group intervention in seven RCTs: In Song's study (Song et al., 2006), 21 patients in the treatment group had myelosuppression of more than a degree, while 24 in the control group. In Chen's study (Chen W. M., 2016), there were 8 cases of bone marrow suppression in the treatment group and 18 cases in the control group. In Dang’s study (Dang and Wang, 2010), there was 1 case of Ⅲ, Ⅳ cardiac function changes in the treatment group and 3 cases in the control group. In Liu's study (Liu et al., 2005), there were only 2 cases of decreased WBC in the control group. The blood image was reduced in 30 cases in the treatment group and 40 cases in the control group, they had statistically significant difference (*p* < 0.05). In the treatment group, individual patients presented with fever, urticaria and other symptoms, which were relieved after symptomatic treatment. In Ye's study (Ye et al., 2002), there were 8 cases of mild drug fever less than 38.5°C. In Ren's study (Ren and Zhang, 2005), the main side effects were nausea and vomiting grade I and II, and decreased WBC. The peripheral hemogram in the treatment group was not significantly reduced, but individual patients developed the symptoms of fever and urticaria. The peripheral hemogram of the control group decreased significantly. In Han’s study (Han et al., 2014), there were 15 cases of myelosuppression in the treatment group and 32 cases in the control group.

SMI combined with CF was used as a treatment group intervention in nine RCTs: In Xiong's study (Xiong et al., 2006), there were 12 cases of ST-T change, 2 cases of QT-QTc extension, 4 cases of sinus tachycardia, 2 cases of QRS low voltage, 3 cases of artrial premature beat, and 1 case of ventricular premature beat in the treatment group, and the incidence was 55.8%. The above symptoms in the control group were 3 cases, 0 cases, 1 case, 1 case, 2 cases, 0 cases, and the incidence was 17.5%. The incidence of electrocardiographic abnormality in the treatment group was significantly higher than that in the control group (*p* < 0.01). In Wang's study (Wang et al., 2012), there were 4 cases of ST-T changes, 3 cases of QT internal prolongation, 1 case of QRS low voltage, 5 cases of nodal tachycardia, 4 cases of premature ventricular beats, 3 cases of ventricular premature contraction, and 2 cases of atrial fibrillation in the treatment group. While in the control group, there were 17 cases, 10 cases, 2 cases, 13 cases, 12 cases, 7 cases, 7 cases respectively. The incidence rates of ST-T changes, QT internal prolongation, nodal tachycardia, premature ventricular beats, and ventricular premature contraction in the treatment group were all lower than that of the control group, their difference was statistically significant (*p* < 0.05 or *p* < 0.01). There was no statistically significant difference between the two groups in the incidence of QRS low voltage and atrial fibrillation (*p* > 0.05). In Yao’s study (Yao et al., 2016), both treatment group and control group had the symptoms of shortness of breath, fatigue, irritability, hot flashes, night sweats, dry mouth and dry throat. However, the scores of TCM symptoms in the treatment group were significantly lower than those in the control group. In Chen’s study (Chen et al., 2018), the adverse reactions of weak, spontaneous sweating, dry mouth and nervous exhaustion were reported. The clinical symptom scores of both groups were lower than those before treatment, and the treatment group was lower than the control group. Zhang’s study (Zhang, 2013) reported bone marrow suppresses gastrointestinal reactions, hair loss, nausea and vomiting, and decreased WBC. In Yang’s study (Yang, 2008), 105 patients in the control group had palpitation, chest distress, nocturnal paroxysmal dyspnea and other discomfort, and one of them had paroxysmal syncope. Only 61 patients in the treatment group had the discomfort of palpitation and chest distress. There were 155 cases of abnormal electrocardiogram in the control group and 109 cases in the treatment group. There were 182 cases of abnormal dynamic electrocardiogram in the control group and 134 cases in the treatment group.

SQFZI combined with CF was used as a treatment group intervention in 16 RCTs: In Liu's study (Liu, 2017), two patients in the treatment group had decreased hemoglobin. While in the control group, five patients had decreased hemoglobin, four patients with liver and kidney poisoning, and two patients had hair loss. The overall incidence of ADRs in treatment group was markedly lower than that in control group. In Zou's study (Zou et al., 2006), according to the ECOG physical condition score, 13, 12, 6, and 0 patients in the treatment group were at grade 0, Ⅰ, Ⅱ and Ⅲ respectively. While there were 3 cases, 6 cases, 8 cases and 14 cases of grade 0-Ⅲ physical condition in the control group. The number of fatigue cases in the treatment group was dramatically lower than that in the control group (*p* < 0.05). Regarding cardiac toxicity, there were 3 cases in Grade Ⅰ, and 10 cases in Grade Ⅱ in treatment group. While there were 4 cases in Grade Ⅰ, 10 cases in grade Ⅱ, and 4 cases in Grade Ⅲ in control group. The number of tachycardia, premature beats, or ST-T changes in electrocardiogram in treatment group was less than that in control group after the second chemotherapy. In Song's study (Song, 2004), there were 2 cases with WBC less than 3.0 × 10^9^ g/L after treatment, 1 case with hemoglobin less than 80 g/L, and 0 cases with PLT less than 60 × 10^9^ g/L. While in the control group, there were 8 cases, 3 cases, and 1 case respectively, and the difference was significant between two groups (*p* < 0.05). In Chen’s study (Chen and Lin, 2007), ADRs were hemotoxicity, non-hemotoxicity and cardiotoxicity. The hemotoxicity was primarily the decrease of WBC, of which the decrease of treatment group was smaller than that of control group and the difference was statistically significant. There was no significant difference in the decrease of PLT and hemoglobin. Non-hemotoxicity reactions caused by chemotherapy drugs included nausea, vomiting, diarrhea, cardiotoxic, stomatitis, and hair loss. The number of fatigue, nausea and vomiting in treatment group was significantly lower than that in control group, and the difference was statistically significant (*p* < 0.05). There were 3 cases of chest distress and palpitation discomfort in the treatment group, while 8 cases in control group, in which 2 cases had paroxysmal dyspnea. In Yang’s study (Yang, 2016), all patients experienced leukopenia, gastrointestinal reactions and heart damage. The incidence of severe toxic and side effects was 25.00% in treatment group and 52.50% in control group, with a significant difference between two groups (*p* < 0.05). In Xie's study (Xie, 2014), there were 12 cases of bone marrow suppression and 2 cases of electrocardiogram changes in treatment group, while 19 cases of bone marrow suppression and 8 cases of electrocardiogram changes in control group. In Xiang’s study (Xiang, 2019), there were 1 case of edema and 1 case of chest pain in treatment group, while 3 cases of edema and 3 cases of chest pain in control group. In Huang’s study (Huang et al., 2008), 70.0% of the patients in treatment group reached level 0 after treatment, while only 43.3% in control group, with statistically significant difference between two groups (*p* < 0.05). In Dai’s study (Dai et al., 2007), the primary ADRs were bone marrow suppression and gastrointestinal reactions. Level Ⅳ side effects were not observed in two groups. In Chen's study (Chen X. C., 2016), there were 10 cases of bone marrow suppression and 1 case of electrocardiogram changes in treatment group, while 20 cases of bone marrow suppression and 6 cases of electrocardiogram changes in control group. In Mi’s study (Mi, 2011), the incidence of degree Ⅲ and degree Ⅳ myelosuppression was 60% in control group, while 16% in treatment group. The increasing trend of transaminase in control group was higher than that in treatment group, and the difference was not statistically significant. There were 7 cases of nausea and vomiting in control group and 19 cases in treatment group. In addition, fever symptoms also existed. In Ma’s study (Ma et al., 2015), there were 2 cases of electrocardiogram changes in treatment group, while 6 cases in control group. In Wang's study (Wang W., 2015), there were 12 cases of decreased hemoglobin in the treatment group and 18 cases in control group. In Fu's study (Fu, 2014), there were 11 cases of decreased hemoglobin in the treatment group and 18 cases in control group. In Wang’s study (Wang et al., 2006), no damage of liver and kidney function was found, and no phlebitis or rash occurred. Only three patients had low fever, which could be relieved by themselves after stoping the medicine.

CKI combined with CF was used as a treatment group intervention in seven RCTs: In Wang's study (Wang and Liu, 2007), there were 4 cases of bone marrow suppression, 0 cases of oral ulcer, and 3 cases of phlebitis in the treatment group. While 18 cases of bone marrow suppression, 5 cases of oral ulcer, and 4 cases of phlebitis in control group. Zhang's study (Zhang and Zhang, 2019) compared the scores of TCM symptoms before and after treatment. The treatment group was superior to control group in terms of fatigue, increased nocturia, lumbago pain, edema, and abdominal distention, and the differences between groups were statistically significant (*p* < 0.05). In Zhai's study (Zhai, 2014), there were 2 cases of oral mucositis in treatment group while 10 cases in control group. In Sun's study (Sun, 2009), only one patient had pain at the site of intravenous drip with flushed complexion, which was recovered after immediate discontinuation. In Ren's study (Ren et al., 2010), the decrease of WBC and abnormal transaminase in the treatment group was better than that in control group. In Wei's study (Wei, 2010), electrocardiogram changes after chemotherapy were observed in two groups, and level Ⅰ and level Ⅱ of toxic reactions in the treatment group were 5 cases and 3 cases respectively, while level Ⅰ, level Ⅱ, and level Ⅲ of toxic reactions in control group were 4 cases, 2 cases, and 2 cases respectively. The main manifestations of cardiotoxicity in two groups were sinus tachycardia and atrial arrhythmia.

HCSI combined with CF was used as a treatment group intervention in one RCT: In Song's study (Song, 2002), the cardiotoxicity in both treatment group and control group was sporadic. Liver and kidney function injuries were also rare, of which the incidence was lower in treatment group (*p* < 0.05). The incidence of venous stimulation was 61.5% in treatment group as well as 23.8% in control group. Patients can recover spontaneously within 3–5 days after drug withdrawal.

HQI combined with CF was used as a treatment group intervention in two RCTs: In Lu's study (Lu, 2010), there were seven patients had decreased PLT in treatment group and 14 patients in control group. There were five patients had abdominal pain in treatment group, while 10 patients in control group. In Huang's study (Huang et al., 2013), both treatment group and control group had 2 cases of level Ⅲ and level Ⅳ platelet decline respectively.

KAI combined with CF was used as a treatment group intervention in 11 RCTs: In Shi's study (Shi et al., 2017), there were 2 cases of decreased PLT and 2 cases of fatigue in treatment group, while 9 cases and 8 cases in control group respectively. In Su's study (Su et al., 2016), there were 14 cases of granulocytopenia in treatment group and 30 cases in control group; 2 cases of fatigue in treatment group and 26 cases in control group; 4 cases of acute diarrhea in treatment group and 8 cases in control group. In Tan's study (Tan et al., 2018), there were 5 cases of thrombocytopenia in treatment group and 21 cases in control group. In Chen's study (Chen F. W., 2016), the incidence of ADRs in the treatment group was 24%, including 1 case of nausea and vomiting, 1 case of abnormal liver function, 1 case of abnormal kidney function, 1 case of abnormal heart function, and 2 cases of WBC decline. The incidence of ADRs in the control group was 60%, including 4 cases of nausea and vomiting, 5 cases of WBC reduction, 2 cases of abnormal liver function, 2 cases of abnormal kidney function, and 2 cases of abnormal heart function. The difference of ADRs of chemotherapy drugs in two groups were statistically significant (*p* < 0.05). In Wu's study (Wu and Wu, 2011), there were 2 cases of abnormal cardiac function in treatment group and 3 cases in control group. In Zhu's study (Zhu and Sun, 2012), the major ADRs were leukopenia and digestive tract reactions, of which the specific number had not been specified.

### Cluster Analysis

**Figure 5:**
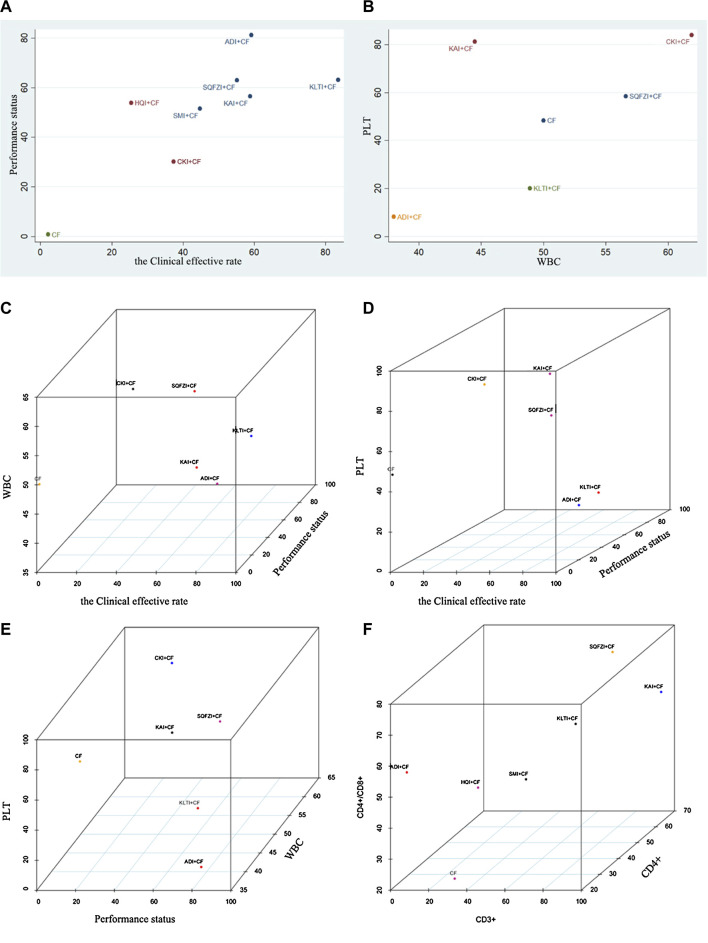
Cluster analysis plot for outcomes. **(A)** Cluster analysis plot of the Clinical effective rate and Performance status; **(B)** Cluster analysis plot of the improvement of WBC and PLT; **(C)** Cluster analysis plot of the Clinical effective rate, improvement of WBC, and Performance status; **(D)** Cluster analysis plot of the Clinical effective rate, improvement of PLT, and Performance status; **(E)** Cluster analysis plot of Performance status, improvement of WBC, and PLT; **(F)** Cluster analysis plot of CD3^+^, CD4+/CD8+, and CD4+. Note: Interventions with the same color belonged to the same cluster, and interventions located in the upper right corner indicate optimal therapy for two different outcomes; SQFZI, Shenqi Fuzheng injection; CKI, Compound Kushen injection; SMI, Shenmai injection; KAI, Kangai injection; ADI, Aidi injection; KLTI, Kanglaite injection; HQI, Huangqi injection; HCSI, Huachansu injection; SI, Shengmai injection; CF, Cyclophosphamide and 5-Fluorouracil.

For primary outcome indicators, cluster analysis was used to evaluate the relative best treatment for BC in this study. Two-dimensional clustering results indicated that ADI and CKI combined with CF, at the position furthest from the zero point, were the best in improving the clinical effectiveness rate and performance status. CKI combined with CF was the preferred treatment to improve the decline of WBC and PLT. Three-dimensional clustering results indicated that SQFZI combined with CF was the preferred treatment in the respect of simultaneously improving the clinical effectiveness rate, performance status, and increasing WBC. KAI combined with CF was the best method to improve the clinical effectiveness rate, performance status, and increasing PLT. CKI and SQFZI combined with CF were both ideal treatments in terms of improving the performance status, increasing WBC, and increasing PLT. SQFZI and KAI combined with CF were both ideal treatments in terms of improving the decline of T cell subsets CD3+, CD4+, and CD4+/CD8+ (Figure 5).

### Publication Bias

**Figure 6:**
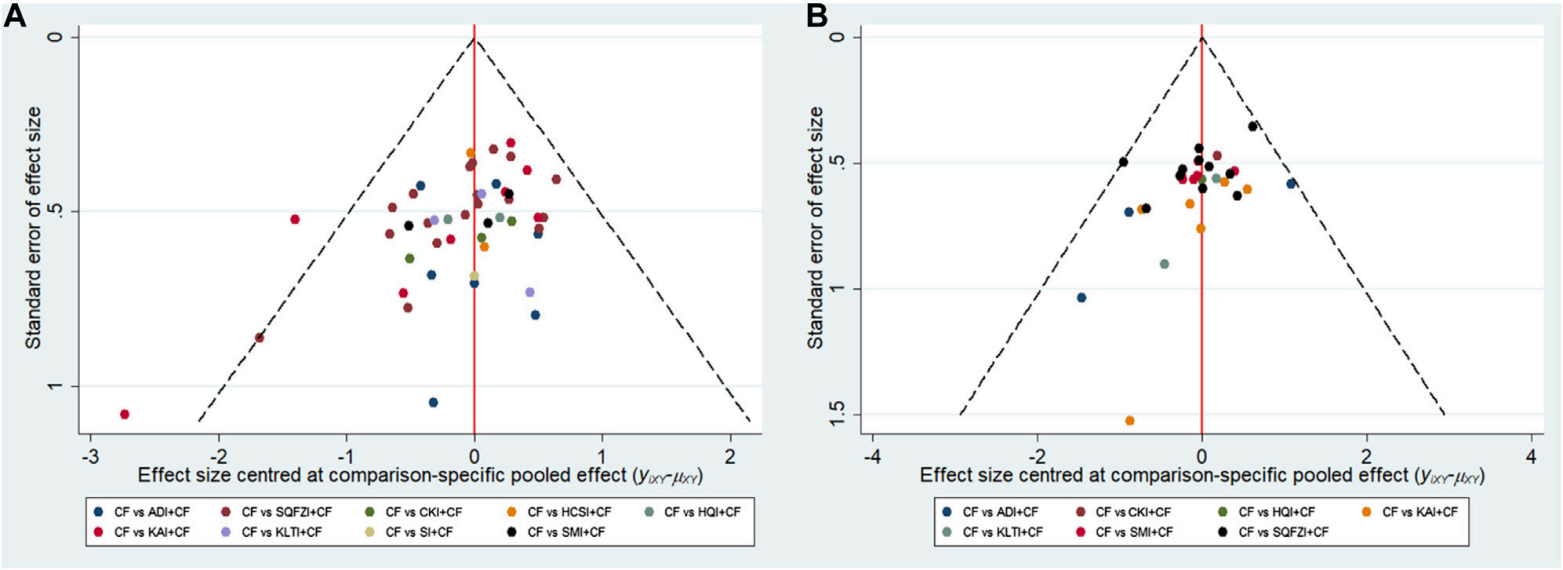
Funnel plots. **(A)** Clinical effectiveness rate; **(B)** Performance status. Note: SQFZI, Shenqi Fuzheng injection; CKI, Compound Kushen injection; SMI, Shenmai injection; KAI, Kangai injection; ADI, Aidi injection; KLTI, Kanglaite injection; HQI, Huangqi injection; HCSI, Huachansu injection; SI, Shengmai injection; CF, Cyclophosphamide and 5-Fluorouracil.

Aiming at the main outcome indicators, this study drawn funnel polts for the clinical effectiveness rate and performance status. Funnel plots of the two outcomes were not completely symmetrical, which indicated that there was a certain publication bias in this study (Figure 6).

## Discussion

Based on the NMA method, this study comprehensively evaluated the efficacy and safety of nine CHIs combined with CF chemotherapy in the treatment of BC. The results of the study indicated that compared with the CF alone, ADI, SMI, SQFZI, KAI, KLTI, and SI combined with CF yielded significantly higher probability of improving the clinical effectiveness rate of BC patients. ADI, SMI, SQFZI, CKI, KAI, and KLTI plus CF were significantly associated with better performance status. The combination of ADI and CF, KAI and CF could more effectively alleviate the reduction of PLT. CF combined with SQFZI could retard the decline of T cell subsets CD3+, CD4+, CD8+, and CD4+/CD8+ in BC patients. With regard to ADRs, CF combined with ADI, SQFZI, and KAI seemed to be the optimal for significantly relieving gastrointestinal reactions. CF combined with SQFZI, CKI, and KAI could better moderate liver and kidney dysfunction. The combined use of CF and KAI was more effective in reducing hair loss symptoms. SMI, SQFZI, HQI, and KAI with CF can relieve leukopenia compared with using CF alone. ADI, SMI, SQFZI, CKI, HQI, and KAI could significantly alleviate the symptoms of nausea and vomiting. Furthermore, the results of cluster analysis suggested that compared with other CHIs, ADI, and CKI with CF for treating BC had its preponderance in improving the clinical effectiveness rate and performance status.

CHIs is a kind of sterile preparation for injection into the human body that is extracted and purified from TCM or prescription Chinese medicine, guided by the TCM theory and using modern technology (National Pharmacopoeia Commission, 2015). Compared with traditional decoction and other oral dosage forms, CHIs has the advantages of rapid onset and high bioavailability, aside from the characteristics of TCM syndrome differentiation treatment (Xu et al., 2018). Some studies have pointed out that ADI can significantly reduce the level of blood CEA and CA199 in patients with gastrointestinal tumors, improve the level of T lymphocyte subgroup, enhance NK cell activity, and improve the performance status of patients (Zhang and Yang, 2009; Deng et al., 2011). The conclusion is consistent with the results of this study. Pharmacological researches have confirmed that SMI contains ginsenoside, Ophiopogon japonicus saponin, ginseng polysaccharide, Ophiopogon japonicus polysaccharide and other active ingredients, which can enhance the anti-tumor ability of tumor patients and have a protective effect on the human hematopoietic system. The mechanism of alleviating leukopenia and enhancing the immune function of patients is related to the role of total ginsenosides in stimulating the proliferation and differentiation of bone marrow hematopoietic stem cells (Feng and Liu, 1995). SQFZI is composed of astragalus and codonopsis with the effects of improving qi deficiency, as well as regenerating and nourishing blood. Astragalus contains various active ingredients such as astragalus polysaccharides, amino acids and glycosides, which can effectively enhance the body's specific immune function and promote the secretion of cytokines. Codonopsis has the function of anti-oxidation and relieving bone marrow toxicity (Wang et al., 2016). CKI has the efficacy of clearing away heat and detoxification, and has a certain inhibitory effect on the growth of tumor cells. The main component, matrine, can inhibit MMP-9 and MMP-2 activation in highly metastatic human breast cancer MDA-MB-231 cells, reduce the mRNA expression levels of MMP-9 and MMP-2, and finally inhibiting tumor cell invasion (Wang, 2018). Studies have shown that CKI can inhibit mouse sarcoma growth by inhibiting ERK, AKT kinase, and BAD phosphorylation (Dai et al., 2019). HCSI is made of toad skin through special processing. Research shows that it can not only significantly improve the phagocytic capacity of macrophages, but also enhance the patient's immunity, reduce bone marrow suppression, peripheral neurotoxicity, gastrointestinal reactions and other adverse effects (Wang and Lu, 2018). The main components of HQI are flavonoids, saponins, amino acids and other secondary metabolites, which have various pharmacological effects, such as enhancing myocardial contractility, increasing the content of superoxide dismutase in the body, scavenging oxygen free radicals, improving microcirculation, lowering blood sugar and blood fat, and regulating immune function (Duan et al., 2017). The main components of KAI are matrine, astragalus, and ginseng. Matrine can inhibit tumors and vascular endothelial cells, and can clear free radicals. Ginsenoside can promote the expression of bone marrow stromal cells, macrophages, and interleukin-6. Astragalus has the effect of regulating and protecting the immune system, renal function, and cardiovascular (Li P P, 2018). KLTI is an injection extracted from coix seed with anti-tumor activity. Modern pharmacological research shows that KLTI can down-regulate Bcl-2 and up-regulate p53 gene expression to inhibit tumor growth and metastasis, adjust the expression of NF-κB, IL-2 and other cytokines, promote T cell proliferation, and improve the body's immunity (Chen et al., 2019). SI is mainly composed of red ginseng, ophiopogon japonicus, and schisandra chinensis. Pharmacological researches have shown that it can inhibit the glycolysis energy supply pathway that tumor cells depend on, enhance the efficacy of drugs, reduce the side effects of chemotherapy, and increase immune cell attack (Hou et al., 2011).

In recent years, the incidence of BC has continued to rise. This is a heavy burden for some developing countries, in which screening and awareness programs are seriously lacking (Nassar et al., 2020). BC is usually called “stone breast” in TCM theory, and in which the pathogenesis of BC is considered to be related to the intermingling of factors such as emotional disorder, Liver-kidney Yin Deficiency, Qi stagnation and blood stasis, and irregular diet (Xie and Long, 2019; Xu and Luo, 2019). CHIs have the functions of improving the internal environment, adjusting the congenital functions, and enhancing the anti-cancer ability of human body. The application of CHIs in clinical adjuvant treatment of cancer is increasingly widespread. So far, only Tian Jinhui’s team has reported a NMA regarding CHIs combined with CF against BC. The study included 26 RCTs, involving 1884 patients and a total of six kinds of CHIs. NMA results showed that CKI and KLTI combined with CF were superior to other programs in improving the clinical effectiveness rate and performance status of patients. SQFZI and ADI combined with CF chemotherapy were more effective in reducing the incidence of nausea and vomiting (grade III to IV) and decreased WBC (grade III to IV). In our study, we added three kinds of CHIs (HQI, SMI and SI) and 58 RCTs, focusing on two new outcomes of T-lymphocyte subsets and peripheral hemogram, meta-analysis results indicated that the evidence is more credible.

At the same time, this study also has several limitations. First of all, the 84 studies included were only discussed the Chinese patients, which caused publication bias to a certain extent. Secondly, the patient's TNM classification and course of disease are different, which may lead to an imbalance between studies. Thirdly, the number of RCTs involved in each CHI is different. For injections with a small sample size, the clinical efficacy of which may be exaggerated, which may bias the results. Finally, this study performed a risk of bias evaluation of the included studies, and the results showed that the quality of them was not very high. A part of the included studies did not specify the specific randomization method, and almost all the studies did not mention the implementation of blinding and allocation concealment. This further affected the credibility of the results of each outcome, and directly led to the low quality of the evidence in this study. Many previous methodological studies have shown that if the design or implementation of clinical trials is flawed, there would be a great risk of causing misleading results, and may even exaggerate the clinical efficacy of interventions. The implementation of blinding and allocation concealment will greatly reduce the bias of the results caused by the subjective ideas of trial participants. Therefore, although the original researches included in this study are RCTs, which are often considered to be the highest quality of evidence, there is limited confidence in the estimated value of the outcome in this study, and its true value may deviate from the estimated value. Given the above limitations, it is recommended that clinical RCTs should be registered in advance according to the Consort standard to ensure the standardization and transparency of the trial process. At the same time, random group hiding and blind methods should be implemented as much as possible to report the test results truthfully. The results of large-sample, multi-center, randomized double-blind controlled trials have higher credibility. Finally, when conducting clinical research on the treatment of BC with CHIs, patients can be diagnosed and treated according to TCM syndromes while adopting western medicine diagnostic criteria to reflect the characteristics of TCM.

## Conclusion

In conclusion, compared with using CF alone, the combination of CHIs and CF was associated with improved treatment performance and safety, and could be beneficial for patients with BC. ADI and KLTI were noteworthy in improving the clinical effectiveness rate and performance status. SQFZI and KAI have better efficacy in reducing ADRs caused by chemotherapy. However, more high-quality clinical RCTs, especially which correctly use blinding and allocation concealment, are required to support the conclusions.

## Glossary

A: Doxorubicin
ADRs: Adverse reactions
ADI: Aidi injection
BC: Breast cancer
C: Cyclophosphamide
E: Epirubicin
F: 5-Fluorouracil
chis: Chinese herbal injections
CIs: Confidence intervals
CR: Complete response
CNKI: The China National Knowledge Infrastructure Database
CKI: Compound Kushen injection
HQI: Huangqi injection
HCSI: Huachansu injection
KAI: Kangai injection
KLTI: Kanglaite injection
KPS: Karnofsky performance score
M: Methotrexate
MDs: Mean differences
NMA: Network meta-analysis
ors: Odds ratios
PLT: Platelet
PR: Partial response
RCTs: Randomized controlled trials
SI: Shengmai injection
SQFZI: Shenqi Fuzheng injection
SMI: Shenmai injection
SUCRA: Surface under the cumulative ranking area curve
T: Pirarubicin
TCM: Traditional Chinese medicine
VIP: The China Science and technology journal Database
WBC: White blood cell
